# A Factorial Randomized Controlled Trial of Implementation-Intention-Based Self-Affirmation Interventions: Findings on Depression, Anxiety, and Well-being in Adults With Psoriasis

**DOI:** 10.3389/fpsyt.2022.795055

**Published:** 2022-03-18

**Authors:** Patryk Łakuta

**Affiliations:** ^1^Institute of Psychology, SWPS University of Social Sciences and Humanities, Warsaw, Poland; ^2^Institute of Psychology, Cardinal Stefan Wyszyński University, Warsaw, Poland

**Keywords:** factorial design, implementation intention, mental health, self-affirmation, RCT—randomized controlled trial, psoriasis, well-being

## Abstract

This study builds on growing evidence on implementation-intention-based self-affirmation intervention effects on mental health. Using a factorial design, this pre-registered study aimed to further investigate whether (1) strengthening the element of specificity within body-related self-affirming implementation intention (BS-AII) intervention compared to general self-affirming implementation intention (S-AII) would provide greater improvements in mental health outcomes for adults with psoriasis, and (2) whether the addition of a booster component would result in enhancing effectiveness at follow-up. A total of 306 adults with psoriasis were assessed for eligibility and 222 (aged 18–71 years) were randomized and received S-AII, BS-AII, or MGI (mere goal intention—control condition). Within each group, participants were again randomized to booster (B) or no-booster condition in a 3 × 2 factorial design, resulting in six groups: S-AII; S-AII + B; BS-AII; BS-AII + B; MGI; and MGI + B. Data were collected over three-time points, at baseline, 2 weeks post-intervention, and at 1-month later. Three primary outcomes were defined as a reduction of anxiety and depressive symptoms and enhancement of well-being. In terms of secondary outcomes, positive other- and self-directed feelings and also an emotional attitude toward the body were evaluated. To fully estimate intervention effects through intention-to-treat analysis, linear mixed models were used. A significant effect of time was observed, but no evidence of time-by-group interactions and no three-way interactions were detected. Exploratory analyses revealed two significant moderating effects of age and self-esteem, pointing to boundary conditions of the interventions. These findings offer to gain deeper insights on null (or negative) effects also reported in past works and highlight that self-affirmation interventions should be more thoroughly investigated and optimized before they can be broadly implemented in real-life contexts, especially to prevent backfiring and negative-enhancing effects.

## Introduction

On a regular basis, people face challenges to their self-concept that could compromise their self-integrity and self-worth. These can range from exposure to threatening messages (e.g., related to individuals' current behaviors such as smoking and alcohol consumption) to being subject to stigma because of a social group belonging/living with a visible skin condition (1–3). Self-affirmation theory (3) hinges on the premise that the effective means of maintaining self-worth and buffering against the threatened domain—e.g., stigma related to a visible skin disease—is to reflect upon domains that are not only different but also positive, valuable, and distinct from the threatened domain, such as personal values, family/friends relationships, accomplishments (e.g., autobiographical recall of mastery events), or special strengths where one feels competent (e.g., being a good worker). The act of affirmation, by broadening self-view, reminds people that the threatened domain is not all that defines their self, and so it mitigates the evaluative implications that a threat to any single identity has on perceptions of the self as a whole [(4); see also (1, 2)]. This provides people with an easy-to-use means of adapting to/rebounding from a threat by enhancing the noticeability of self-resources and placing the threat within a larger context. A broader view of the self that emphasizes a sense of being generally good and valued is released.

Self-affirmation contributes to the section of methods that can help people in restoring self-integrity and lowering distress when facing stressful or threatening events (3, 5, 6). Over the past three decades, much effort and time have been devoted to self-affirmation research to document its beneficial effects, especially in educational settings [e.g., (7); see also (5, 6)]. Self-affirmation has also shown its effectiveness in the health domain and disease prevention [see (8)]. Moreover, in the past several years, findings have supported its potential in mental health improvement in high-risk populations [c.f. (9–11)]. Nevertheless, so far only a relatively few studies have directly focused on the effects of self-affirmation on mental health and well-being, and overall, the results are inconclusive. Some point to no effects or even negative ones [c.f. (12, 13)], while others report improvement of both mental health outcomes and well-being following self-affirmation-based interventions [c.f. (9, 11, 14–17)]. Given promising findings reported in past years in this field, self-affirmation framed as a mental health intervention merits further investigation.

The present study builds on growing evidence [e.g., (9, 15–17); see also (10)] on the effects of implementation intention (II)-based self-affirmation intervention on mental health outcomes, including its effectiveness evaluated in adults with psoriasis (11), a highly-stigmatized skin condition associated with elevated risk for depression, anxiety, and lower levels of well-being. Using a factorial design, this pre-registered study aimed to further investigate whether (1) body-related self-affirming implementation intention (BS-AII), with augmented specificity of the intervention compared to general self-affirming implementation intention (S-AII), would provide greater improvements in mental health outcomes for adults with psoriasis; and (2), whether the addition of a booster component would result in enhancing effectiveness—greater reduction in depression and anxiety and more increase of well-being at follow-up. As standard comparative RCTs have limitations for determining the specific contribution of individual components within a psychological intervention package and for inferring causality concerning their mechanisms (18, 19), this study adopted a full factorial design having advantages of directly testing individual components and their interactions, being able to distinguish specific factors from common factors, and being more efficient and economical with respect to sample size and resources.

### If-Then Plans With Self-Affirming Cognitions as an Effective Means of Affirming the Self at Experiencing Psychological Threat

Self-affirmation (3) is an act of reflecting upon one's cherished values, important relationships, positive traits, or recalling accomplishments to restore/sustain one's perception of adequacy. As shown in past research (5, 6, 10), it may be seen as a flexible process, executed through a variety of means, with a quite diffuse impact, can affect and promote more adaptive reactions in response to a variety of self-threats. Typically, researchers in this area ask participants to affirm their core values or positive personal characteristics using various written tasks [c.f. (20)]. In the past few years, however, studies have demonstrated the beneficial effects of combining self-affirming cognitions with the implementation intention paradigm (21), creating a new means of self-affirming known as self-affirming implementation intention (S-AII) [(22); see also (9, 15–17)]. In this standardized self-affirmation intervention, participants are asked to formulate an if-then plan with one preferred self-affirmation-inducing cognition (e.g., “*If* I feel sad, threatened, or uneasy by something, *then* I will think about the things I value about myself”) [c.f. (22)]. Such ultra-brief intervention that can be widely distributed, readily self-applied outside of clinical settings, with no clinical supervision required, may better suit the needs of people than an effort- and time-demanding expressive writing (e.g., an essay on cherished values). Most importantly, the if-then structure of implementation intentions makes self-affirming cognitions accessible once a person's self-system is threatened. Consequently, the act that involves focusing on important and valuable aspects of the self to restore or sustain one's perception of adequacy can proceed and be timely.

Implementation intentions are a self-regulatory strategy that helps to translate any kind of plan into action and thus, can be adjusted to the various challenges in daily life. Goal intention (“I want to achieve X/perform behavior X!,” with X representing desired future, outcome, or behavior) alone does not ensure the action, and success especially; goal setting should be followed by planning [c.f. (21)]. An implementation intention—*if-then* plan—specifies when, where, and how one wants to perform goal-directed responses such as instrumental thoughts, feelings, or actions that help to realize the formed goal intention (i.e., *if* situation Y arises, *then* I will perform goal-directed response Z to achieve goal X) (21). Importantly, implementation intentions strengthen the links between the critical situation and the goal-directed response and promote accessibility of situation cues so that, upon encountering the relevant cues, the goal-relevant response would appear automatically (23, 24). By enabling people to automatically initiate the planned response once they encounter the specified situation, implementation intentions narrow the gap between goal and needed action and optimize goal pursuit [for a review, see (25)]. In that vein, a self-affirming implementation intention is a specific mode-of-thought-inducing plan (i.e., self-affirmation-thought-inducing) developed to address mental health and emotion regulation issues. Besides, relative to expressive writing, this approach offers an easier and handy to implement technique that is better conformed on a daily basis activity, outside an experimental setting [(22); see also (9, 11, 15–17)].

### Effects of Self-Affirming Implementation Intentions on Mental Health and Well-Being

Past self-affirmation works have primarily focused on testing self-affirmation as a means of defensiveness reduction toward threatening stimuli or messages, mostly in health, education, and relational contexts [c.f. (5, 6)]. Recently, researchers more boldly have been testing self-affirmation outside the area of health behaviors and education. Especially, studies on mental health and well-being effects of S-AII have demonstrated promising findings. For example, there has been a significant reduction in social anxiety symptoms (*d* = −0.42) during a 2-week intervention based on S-AII (15). It has also been shown that the intervention can be used to protect subjective well-being in a community sample of older women (9), can reduce work-related anxiety in downsizing survivors (17), and furthermore, the application of S-AII may be of benefit to the well-being of teachers and other highly stressed workers (16). These findings corroborate the notion that self-affirmation might lead to positive outcomes beyond the maintenance of a more favorable self-image. Notably, recent research has shown positive short-term effects (i.e., over 2 weeks) on a range of mental health indices, including depression (*d*s: −0.40 to −0.55), anxiety (*d*s: −0.45 to −0.60), and well-being (*d*s: 0.25–0.41) in adults with psoriasis, subsequent to II-based self-affirmation intervention (11). These findings are quite impressive given that the intervention conducted in the high-risk community was brief and low in intensity, and the intervention effects were not augmented by the inclusion of any booster component. It is also notable that the intervention effects were compared with both passive and active comparison groups, providing more robust evidence for the potency of S-AII. Despite these promising findings, however, significant differences between the groups faded at 1-month follow-up. Broadly, past research has only reported short-term S-AII effects. Therefore, at this stage of self-affirmation research, longer maintenance of effects post-intervention and selection of planned methods for such maintenance are key issues to address.

Maintenance of effects beyond intervention completion is an important requisite to judge a valid intervention as more than a temporary shift in symptom level. Naturally, one of the possible methods for maintaining the effects of the intervention for a longer term is the inclusion of a booster component. Boosters have been valued in mental health treatment and therapeutic interventions for several decades; however, they may have inconsistent and/or modest importance in maintaining effects [see e.g., (26)]. Careful consideration and rigorous testing in factorial design research are thus necessary before much more than speculations can be made about what maintenance of intervention effects or lack thereof means, whether including a booster or not. This research aims to empirically test the relative effectiveness of S-AII intervention with and without a booster component.

There is also another potentially impactful approach for the enhancement of II-based self-affirmation intervention. Recently, a crucial hint has emerged from mediational research of self-affirmation effects. Findings on indirect effects can help in informing further development of the intervention, basically in the manner of what active ingredients should be intensified and refined toward the most favorable results. It has been shown that cognitive regulatory processes along with positive self-oriented feelings in the orderly sequence appeared to mediate the association between the S-AII intervention and mental health outcomes [Łakuta^1^; see also (27)]. Specifically, the S-AII effects on depression, anxiety, and well-being were driven by improving cognitive regulatory processes (i.e., improving cognitive emotion regulation as decreasing the usage of catastrophizing/enhancing the usage of positive refocusing) and enhancing positive self-oriented feelings, including lowering levels of negative body-related feelings. Given negative emotions in self aspects are among key risk factors of negative mental health outcomes (28–30) and negative body-related feelings have been recognized as a fundamental target for interventions among adults with psoriasis [e.g., (31–33)], these indirect effects seem particularly noteworthy. These findings point to potential ways in which S-AII interventions can be modified by targeting body-/skin-related issues to strengthen the element of specificity of the intervention. As such, it is worth formally testing whether a critical situation in the *if*-part of if-then plans with self-affirming cognitions being specifically defined to body-related issues could further strengthen their effectiveness. Regarding community adults with psoriasis, a specific mode of body-issue-related plan may promote recognition of the most relevant situation cues, enabling more adjusted self-regulation and higher effectiveness of self-affirming thoughts. Therefore, the proposed modification of the standard S-AII called *body-related self-affirming implementation intention* (BS-AII) was tested in the current study.

### The Present Research

This pre-registered study^2^ was designed to compare the effectiveness of *body-related self-affirming implementation intention* (BS-AII) relative to (standard) *self-affirming implementation intention* (S-AII) and control condition, with forming *mere goal intention* (MGI). A direct comparison between BS-AII vs. S-AII could provide information on whether it is the focus on a specific area rather than on more general threats fosters beneficial effects. Moreover, the second aim of the study was to establish the effects of a booster component as a strategy for optimizing beneficial longer-term effects of II-based self-affirmation interventions, predicting that the intervention conditions augmented with a booster session would exert better mental health outcomes assessed at follow-up (i.e., 1 month after the second point of the study). Especially, it was predicted that giving participants the opportunity to form again BS-AII/S-AII at T2 (i.e., at 2 weeks post-intervention) would extend the effects of the initial intervention to the final study point. In light of the results of prior research on forming II [c.f. (34, 35)], it was hypothesized that this would act as a reminder/booster, enabling to maintain the benefits that are demonstrated in the short-term self-affirmation interventions [but appear to diminish over time, see e.g., (11, 15)]. The booster session may allow participants to either recall and repeat the original if-then plans with self-affirming cognitions or make changes in their plans with other self-affirming cognitions that provide more suitable alternatives. As a result, the reminder may just serve to sustain the initial change (as it is hypothesized) or even create an augmented effect to further promote mental health benefits over and above the change demonstrated post-intervention, hence vividly improving long-term effectiveness.

Three primary outcomes were defined as a reduction of anxiety and depressive symptoms and enhancement of well-being. In terms of secondary outcomes, positive other- and self-directed feelings and also an emotional attitude toward the body were evaluated. It was hypothesized that the tested self-affirmation approaches would result in different levels of effectiveness both on primary and secondary outcomes. The intervention conditions adopting S-AII and BS-AII were hypothesized to result in statistically significant independent improvements in the outcome measures relative to the MGI condition: (i) 2 weeks post-intervention and (ii) at the follow-up 1-month later. The BS-AII, however, as having more specificity, was predicted to be more successful. It was also expected that the II-based self-affirmation intervention conditions with a booster component would lead to greater change in outcomes than the S-AII or BS-AII alone. Finally, it was also hypothesized that the BS-AII intervention with the booster component would outperform, compared with the other study conditions.

Furthermore, at this stage of S-AII research, it is essential to put intensified efforts to gain a clear understanding of factors that can explain for whom self-affirmation works best, and what active intervention ingredients/circumstances could be enhanced/refined to yield the most favorable and powerful effects. Recently, researchers have growingly recognized the importance of documenting systematically the circumstances under which self-affirmation yields positive, no, or even negative effects [c.f. (5, 8, 36); Łakuta (see text footnote 1, respectively)]. For example, Ferrer and Cohen (8) in the health behavior domain have emphasized the importance of the availability of resources, the presence of threat, and the timeliness of self-affirmation with respect to threat and resources, so that self-affirmation can show its assumed (and beneficial) effects on outcomes. Recent research on self-affirmation effects on mental health seems to corroborate this notion Łakuta (see text footnote 1, respectively). In regard to psychological threat, it has been found that the presence of higher social stigmatization significantly increased the effects of S-AII intervention. More beneficial effects of S-AII in terms of reduction of anxiety and depressive symptoms were observed in adults with psoriasis experiencing moderate and high levels of stigmatization. In the realm of contemporary self-affirmation research, *for whom does it work* is one of the crucial questions, which undoubtedly cannot be missed out. Delineating boundary conditions for the presence or absence of self-affirmation effects by testing moderating influences is crucial to advancing our understanding either of when or for whom but also how self-affirmation works. Consequently, in the current study, to aid the development of self-affirmation theory, moderators of the intervention effects were explored. The choice of moderator variables was guided by recent literature on self-affirmation and findings concerning risk factors for mental health problems in adults with psoriasis, reviewed above. Several putative moderators were tested, i.e., socio-demographic characteristics, disease severity, social stigmatization, and level of dispositional resources—self-esteem (all assessed at baseline, before randomization). However, as these analyses do not pertain to the registered hypotheses (i.e., the confirmatory part of the work), they should be considered exploratory.

## Materials and Methods

### Participants

**Figure 1:**
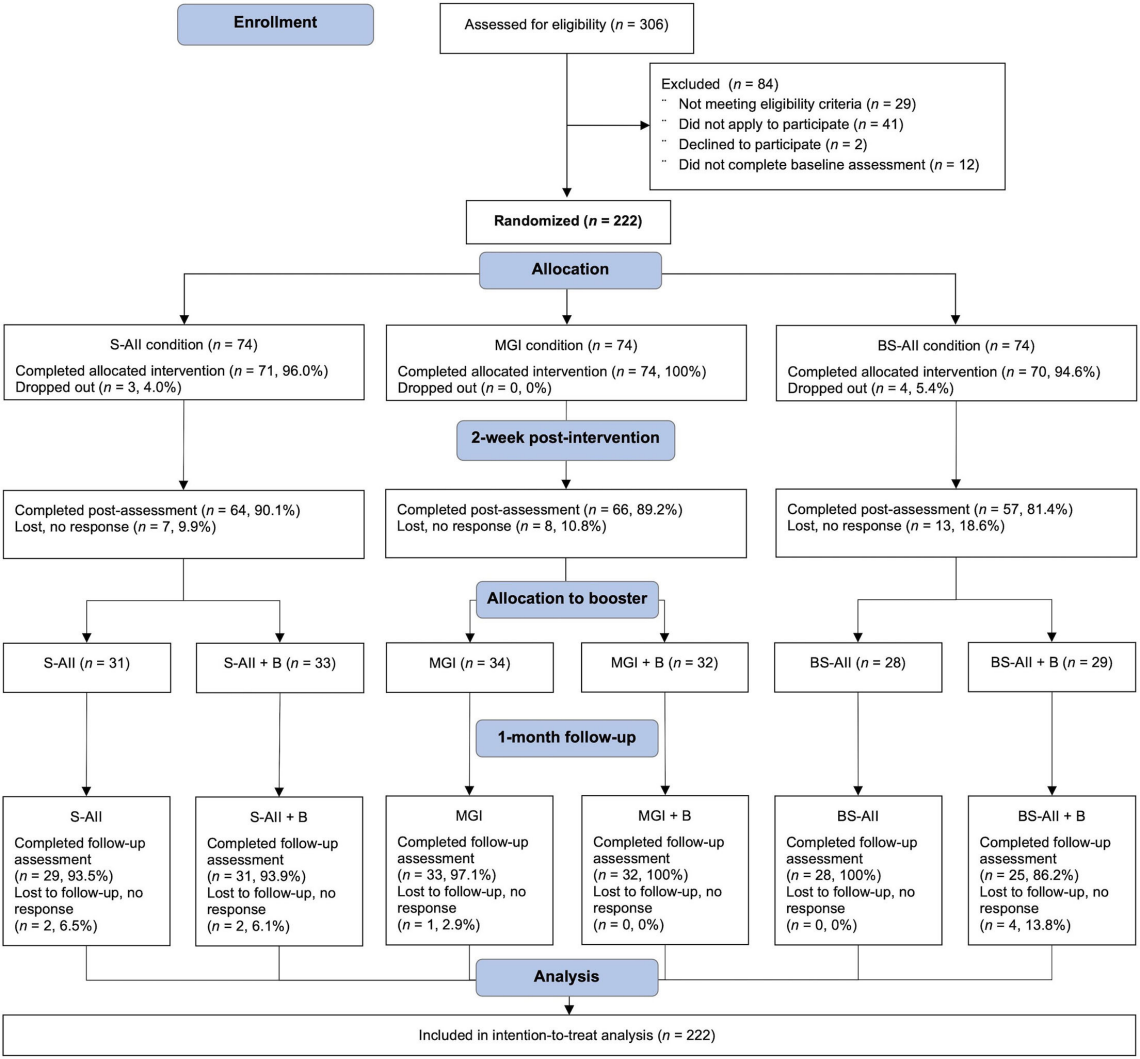
CONSORT flow diagram: enrollment, group randomization, attrition, and data analysis.

Figure 1 graphically presents the design of the study, including enrolment, intervention, follow-up, and data analysis. Participants in the study were 222 adults with psoriasis aged 18–71 years (*M*_age_ = 33.86 years, *SD*_age_ = 10.36). Of the individuals in the randomized sample, the majority were female (86.0%), married/cohabited with a partner (72.1%), had paid employment (74.8%), were highly educated (58.1%), and with the mean psoriasis severity of 9.01 SAPASI score (*SD* = 7.22, range: 0–38; 5.4% of the participants were in remission and 34.2% with moderate or severe psoriasis, i.e., score ≥10). Plaque psoriasis made up 75.7% of the cases in the sample, and scalp psoriasis was the second most common form in the sample (55.4%). Only 6.0% of the participants were receiving biological treatment.

Participants were recruited through an online advertisement (social media, e.g., Facebook) as well as a series of offline methods (i.e., flyers and posters in psoriasis patient associations, hospitals, and outpatient clinics) and screened before entering the trial. Individuals were considered eligible for participation if they (a) were 18 years old or older, (b) had physician-diagnosed psoriasis, (c) had internet access and had a valid e-mail address, and (d) read and accepted the informed consent. Exclusion criteria were the following: participating in other psychosocial or pharmacological treatments or being enrolled in a trial or in any research on mental health (e.g., a clinical trial of an investigational medical product).

### Study Design and Implementation

The study was pre-registered on the Open Science Framework (see text footnote 1) before any data were collected and approved by the University's Human Research Ethics Committee. The study is based on a 3 × 2 factorial design, testing two experimental factors: intervention (three levels: *mere goal intention* vs. *self-affirming implementation intention* vs. *body-related self-affirming implementation intention*) and booster component (two levels: *no* vs. *yes*). Participants were randomized across these two factors. The randomization to the study conditions was conducted by the Qualtrics randomizer feature. A total of 222 adults were randomized and received S-AII, BS-AII, or MGI intervention. Within each group, participants were again randomized to booster (B) or no-booster condition (see Figure 1), enabling a full parallel-group design, with six conditions reflecting all of the possible combinations: (1) forming mere goal intention (MGI); (2) self-affirming implementation intention (S-AII); (3) body-related self-affirming implementation intention (BS-AII); (4) mere goal intention with booster component (MGI + B); (5) self-affirming implementation intention with booster component (S-AII + B); and (6) body-related self-affirming implementation intention with booster component (BS-AII + B).

All measurements and intervention materials were delivered using the Qualtrics software. The study information sheet provided participants with information that they would be asked (besides questionnaires on mood, the disease, and coping with psoriasis) to complete with a written task concerning their functioning in the following weeks, without specifying conditions, thus leaving them unaware of the specific group assignment at the time of the study. Moreover, as randomization to the groups was conducted automatically, the research team was also blinded to allocation. Data were collected over three time points: at baseline (time 1, T1), 2 weeks post-intervention (time 2, T2), and at 1-month later (time 3, T3). As an incentive to participate and in order to deter attrition, a voucher of about $25 was given to participants who completed all measurements. A sample size of 168 (28 per group) was estimated by *a priori* power analysis for detecting a small effect (Cohen's *f* = 0.10) in a mixed model analysis of variance with six conditions and three points of measurement (i.e., baseline to follow-up, T3), with power set to 80%, a significance level of 5%, and a correlation of 0.7 between measures. Allowing a dropout rate up to 30%, a sample of 222 would be needed (and that exact sample size was adopted as a minimum at baseline).

### Outcome Measures

The three pre-registered primary outcomes involve depression, anxiety, and well-being levels. Anxiety severity was measured by the 7-item Generalized Anxiety Disorder scale [GAD-7; (37)]. Depression severity was measured by the 9-item Patient Health Questionnaire [PHQ-9; (38)]. Well-being, conceptualized as involving both hedonic and eudaimonic aspects, was measured with the 14-item Mental Health Continuum–Short Form [MHC-SF; (39, 40)]. Overall, all scales had good to excellent internal consistency reliability across all study points: alpha coefficient for scale scores at baseline ranged from 0.87 to 0.94 (*M*_α_ = 0.90). Similarly, at T2 and T3, alpha coefficient for scale scores ranged from 0.87 to 0.95 (*M*_α_ = 0.91).

Secondary outcome measures included changes in terms of (i) positive self-directed feelings, (ii) positive other-directed feelings, and (iii) emotional attitude toward the body. Positive other- and self-directed feelings were measured by asking participants to indicate how often they have experienced five prosocial (e.g., love, empathic, connected, and grateful) and positive feelings directed toward themselves (e.g., pride, feeling strong, and in control) in their daily lives, respectively (41, 42). Emotional attitude toward the body was measured by the nine-item Body Emotions Scale (43), with higher sum scores reflecting more negative body emotions. All these scales had good to excellent internal consistency reliability across all study points: alpha coefficient for scale scores at baseline ranged from 0.82 to 0.90 (*M*_α_ = 0.86). Similarly, at T2 and T3, alpha coefficient for scale scores ranged from 0.80 to 0.94 (*M*_α_ = 0.87).

### Potential Moderators

Multiple baseline characteristics were explored as potential moderators of the intervention effects, including sociodemographic characteristics (i.e., age, gender, marital status, and educational level), the disease severity [based on the Self-Administered Psoriasis Area and Severity Index—SAPASI; (44, 45)], exposure to stigma due to psoriasis [measured by the Stigmatization Scale; (46)], and dispositional resources—self-esteem measured using Robins et al.'s (47) single-item self-esteem scale.

### Interventions

#### Mere Goal Intention (MGI) Condition

Participants in the MGI group received instruction to merely identify and form a goal intention regarding adaptive functioning and feeling good in the next weeks (i.e., an intention in the format “I want to achieve X/perform behavior X!,” with X representing desired future, outcome, or behavior) [see (21)]. Examples of participants' goals that were set: “I want to keep my worries under control;” “I want to start working out;” “I want to spend more time with my family.” The condition was chosen as the basic comparator to allow the effect of the self-affirmation interventions to be examined above and beyond the effect of simply setting goal intention.

#### Self-Affirming Implementation Intention (S-AII) Condition

The S-AII is a brief standardized self-affirmation intervention in which participants are asked to form an if-then plan with self-affirming cognitions, for example: “If I feel sad, threatened, or uneasy by something, then I will think about the things I value about myself” [c.f. (22); see also (16, 17)]. Thus, the intervention employs a self-affirm paradigm but also makes use of the if-then structure of implementation intentions (21). Participants were provided with the implementation intention prompt in the form of a sentence stem: “If I feel sad, threatened or uneasy by something, then I will…,” where “feeling sad, threatened, or uneasy by something” is the critical situation; and a choice of appropriate self-affirming responses, with which to complete the sentence, included six options representing a focus on personally important values, strengths/attributes, and social relationships, e.g., “…think about my values;” “…think about the things I value about myself;” “…remember things that I have succeeded in;” and “…think about the people who are important to me,” adapted from Harris et al. (48). Participants were asked to point the preferred response using the checkbox and type out the stem and their chosen option on three blank lines. In order to encourage participants to type the self-affirming sentence out in full, they were prompted with “*If* …” at the beginning of the first blank line. Afterward, participants were asked to read the plan three times and to repeat it silently to themselves.

#### Body-Related Self-Affirming Implementation Intention (BS-AII) Condition

In the BS-II, to strengthen the element of specificity of the intervention, a critical situation in the *if*-part of if-then plans is designed to be specifically defined to body issues and then linked explicitly to self-affirming responses. Participants were given the same set of self-affirming responses as in the S-AII condition, with which to complete their implementation intentions, but with the modified sentence stem, that is “*If I feel sad, threatened or uneasy about my appearance and my skin condition, then I will*….” Similar to the S-AII condition, participants were asked to point the preferred response using the checkbox and type out the stem and their chosen option on three blank lines.

#### Booster Component

Participants randomized to the booster conditions after the T2 assessment were presented with the same intervention materials as at the first session of the intervention. They were asked to form S-AII/BS-AII (or MGI) for the next weeks. Basically, the provision of the “booster” planning session served to enable participants to either repeat the original plans/goals (and enhance their recall) or form another plan/goal that is a more suitable alternative [c.f. (34, 35)].

### Statistical Analysis

Primary analyses were conducted using an intention-to-treat approach, including all participants who had been randomized. Six linear mixed models (LMMs) were tested comparing intervention and control groups on primary outcome measures (depressive and anxiety symptoms, and well-being) and secondary outcome measures (positive other- and self-directed feelings, and emotional attitude toward the body). LMM has superior qualities as accounting for natural correlation between repeated measurements, handling missing values, and the use of all available data, making this a full intention-to-treat analysis. All models were fitted with maximum-likelihood estimation and an unstructured covariance matrix [c.f. (49)], each included a random factor for subjects to account for correlation among repeated measures. In all models, time, intervention condition, booster, and their interactions were included as fixed factors. Covariates were age and sex. Age was included because older patients with psoriasis have been shown to be at increased risk for mental health issues [e.g., (50)]. Sex was included because the resulting sample was not gender-balanced and also to regard gender differences in mental health problems that have been reported in adults with psoriasis [e.g., (32, 33); see also (50)].

For exploratory purposes, moderation analyses were conducted using PROCESS macro version 3.5.3 (51). All baseline characteristics were explored to test potential moderators of the intervention effects in single moderation models for each study outcome, with a categorical independent variable based on Helmert coding of groups [see (52)]. For example, about post-intervention data, considering a multicategorical group variable with *k* = 3 categories, there are two constructed variables *D*_1_ and *D*_2_, enabling for MGI (control group) to be contrasted to both BS-AII and S-AII (first interaction term), and S-AII to be directly contrasted to BS-AII (second interaction term), generating comparisons of interest in this study.

## Results

### Randomization Check and Attrition Analysis

A series of ANOVA and chi-squared tests indicated that at baseline the study arms did not significantly differ regarding sociodemographic characteristics, the disease severity, and outcome measures as well (all *p*-values >0.101), indicating successful randomization (see Table 1).

**Table 1:** Baseline characteristics of the participants (*N* = 222).

	**S-AII**	**MGI**	**BS-AII**	**Test statistics**
	**(*n* = 74)**	**(*n* = 74)**	**(*n* = 74)**	
**Demographics**				
Age (years), *M* (*SD*)	31.77 (9.06)	34.69 (10.64)	35.12 (11.08)	*F*_(2, 219)_ = 2.32, *p* = 0.101
Gender, *n* (%)				$\chi_{\left(2,N=222\right)}^{2}$ = 0.08, *p* = 0.963
Female	63 (85.1%)	64 (86.5%)	64 (86.5%)	
Male	11 (14.9%)	10 (13.5%)	10 (13.5%)	
Marital status, *n* (%)				$\chi_{\left(2,N=222\right)}^{2}$ = 1.93, *p* = 0.382
Married/cohabiting	55 (74.3%)	56 (75.7%)	49 (66.2%)	
Not married (single, divorced, widowed)	19 (25.7%)	18 (24.3%)	25 (33.8%)	
Education level (highest level completed), *n* (%)				$\chi_{\left(4,N=222\right)}^{2}$ = 3.81, *p* = 0.432
Low (primary school, lower secondary)	2 (2.7%)	2 (2.7%)	0 (0.0%)	
Intermediate (upper secondary education)	30 (40.5%)	27 (36.5%)	32 (43.2%)	
High (tertiary education, university degree)	42 (56.8%)	45 (60.8%)	42 (56.8%)	
Work status, *n* (%)				$\chi_{\left(6,N=222\right)}^{2}$ = 8.77, *p* = 0.187
Student	10 (13.5%)	6 (8.1%)	8 (10.8%)	
Paid employment	55 (74.3%)	56 (75.7%)	55 (74.3%)	
Unemployed	9 (12.2%)	7 (9.4%)	7 (9.5%)	
Pensioner or retired	0 (0.0%)	5 (6.8%)	4 (5.4%)	
Psoriasis severity (SAPASI), *M* (*SD*)	8.72 (7.96)	9.01 (7.11)	9.30 (6.62)	*F*_(2, 219)_ = 0.12, *p* = 0.888
**Primary outcomes**				
PHQ-9, *M* (*SD*)	10.55 (5.63)	10.28 (6.11)	9.18 (6.20)	*F*_(2, 219)_ = 1.10, *p* = 0.334
Mild symptoms (PHQ-9 ≥ 5–9), *n* (%)	27 (36.5%)	26 (35.1%)	25 (33.8%)	$\chi_{\left(2,N=222\right)}^{2}$ = 1.31, *p* = 0.521
Prevalence of depression (PHQ-9 ≥ 10), *n* (%)	36 (48.6%)	36 (48.7%)	30 (40.5%)	$\chi_{\left(2,N=222\right)}^{2}$ = 1.31, *p* = 0.521
GAD-7, *M* (*SD*)	11.04 (5.77)	11.00 (5.36)	9.81 (5.60)	*F*_(2, 219)_ = 1.16, *p* = 0.315
Mild symptoms (GAD-7 ≥ 5–9), *n* (%)	28 (37.8%)	25 (33.8%)	30 (40.5%)	$\chi_{\left(2,N=222\right)}^{2}$ = 0.73, *p* = 0.694
Prevalence of anxiety (GAD-7 ≥ 10), *n* (%)	37 (50.0%)	42 (56.8%)	33 (44.6%)	$\chi_{\left(2,N=222\right)}^{2}$ = 2.20, *p* = 0.333
MHC-SF (total score), *M* (*SD*)	29.82 (13.07)	32.35 (15.26)	31.73 (17.08)	*F*_(2, 219)_ = 0.55, *p* = 0.576
MHC-SF EW, *M* (*SD*)	7.04 (3.45)	7.36 (3.54)	7.24 (4.02)	*F*_(2, 219)_ = 0.15, *p* = 0.864
MHC-SF SW, *M* (*SD*)	7.85 (4.91)	8.39 (5.96)	8.57 (6.49)	*F*_(2, 219)_ = 0.30, *p* = 0.738
MHC-SF PW, *M* (*SD*)	14.93 (6.81)	16.59 (7.22)	15.92 (8.22)	*F*_(2, 219)_ = 0.93, *p* = 0.395

*S-AII, self-affirming implementation intention condition; MGI, mere goal intention condition (control condition); BS-AII, body-related self-affirming implementation intention condition; SAPASI, Self-Administered Psoriasis Area and Severity Index (the range of absolute SAPASI scores is 0 – 72); PHQ-9, 9-item Patient Health Questionnaire; GAD-7, 7-item Generalized Anxiety Disorder Scale; MHC-SF, Mental Health Continuum–Short Form; EW, emotional well-being subscale; SW, social well-being subscale; PW, psychological well-being subscale*.

At post-intervention and at 1-month follow-up, data were available for 187 and 178 participants, respectively. As seen in the trial flowchart (Figure 1), data attrition was fairly low at both the primary (15.8%) and secondary (4.8%) endpoint. The overall attrition rate was 19.2%. Of note, attrition between randomization and completion of post-intervention and follow-up assessments was found to not differ across the study conditions (all *p*-values >0.321), indicating that the dropout was non-systematic.

### Intervention Effects on Primary and Secondary Outcomes

LMM analyses, for depressive and anxiety symptoms and well-being, controlling for age and gender as covariates, revealed a significant effect of time but non-significant effects of condition, booster, and their interactions (Table 2). Overall, significant time effect showed that mental health outcomes continued to improve significantly across groups at post-test (T2) and at 1-month follow-up (T3) but there were no significant group differences between at both time points. Estimated means are presented in the Supplementary Material.

**Table 2:** Results of the LMM analyses for primary outcomes, omnibus tests for fixed effects.

	**Depression**				**Anxiety**					**Well-being**		
	***F***	***df*_1_**	***df*_2_**	***p***	***F***	***df*_1_**	***df*_2_**	***p***	***F***	***df*_1_**	***df*_2_**	***p***
Time	11.91	2	377	<0.001	30.82	2	380	<0.001	11.94	2	372	<0.001
Condition	1.48	2	219	0.229	0.892	2	217	0.412	0.35	2	220	0.702
Booster	0.01	1	220	0.995	0.04	1	218	0.844	0.02	1	220	0.895
Gender	0.99	1	226	0.322	0.39	1	226	0.534	1.26	1	224	0.262
Age	1.49	1	217	0.224	1.86	1	215	0.174	0.11	1	218	0.740
Time × condition	0.12	4	377	0.978	1.41	4	380	0.230	0.19	4	372	0.942
Time × booster	0.23	2	377	0.791	1.08	2	380	0.341	1.53	2	372	0.219
Condition × booster	0.49	2	219	0.613	0.21	2	218	0.815	0.01	2	220	0.994
Time × condition × booster	0.73	4	377	0.574	0.15	4	380	0.965	1.50	4	372	0.200
	*R*^2^ marginal	0.04			0.07				0.02			
	*R*^2^ conditional	0.66			0.58				0.77			

*df, degrees of freedom (Satterthwaite method for degrees of freedom)*.

A similar pattern was observed for secondary outcomes (Table 3). The LMM analyses, controlling for age and gender, revealed a significant effect of time but non-significant effects of condition, booster, and their interactions.^3^

**Table 3:** Results of the LMM analyses for secondary outcomes, omnibus tests for fixed effects.

	**Emotional attitude toward the body**				**Positive self-directed feelings**				**Positive other-directed feelings**			
	***F***	***df*_1_**	***df*_2_**	***p***	***F***	***df*_1_**	***df*_2_**	***p***	***F***	***df*_1_**	***df*_2_**	***p***
Time	24.30	2	375	<0.001	21.05	2	374	<0.001	0.28	2	378	0.754
Condition	0.55	2	222	0.577	0.18	2	218	0.840	0.69	2	219	0.502
Booster	0.90	1	222	0.343	0.16	1	218	0.688	0.10	1	219	0.754
Gender	0.20	1	226	0.657	1.52	1	223	0.219	9.38	1	226	0.002
Age	0.01	1	220	0.928	0.01	1	216	0.983	2.04	1	217	0.154
Time × condition	1.70	4	375	0.149	1.22	4	374	0.301	0.63	4	378	0.641
Time × booster	1.00	2	375	0.370	1.45	2	374	0.234	0.02	2	378	0.978
Condition × booster	0.37	2	222	0.694	0.36	2	218	0.702	2.50	2	219	0.085
Time × condition × booster	1.95	4	375	0.102	2.28	4	374	0.060	0.55	4	378	0.701
	*R*^2^ marginal	0.04			0.05				0.07			
	*R*^2^ conditional	0.76			0.70				0.65			

*df, degrees of freedom (Satterthwaite method for degrees of freedom)*.

### Exploratory Analysis: Testing Moderation Effects

**Figure 2:**
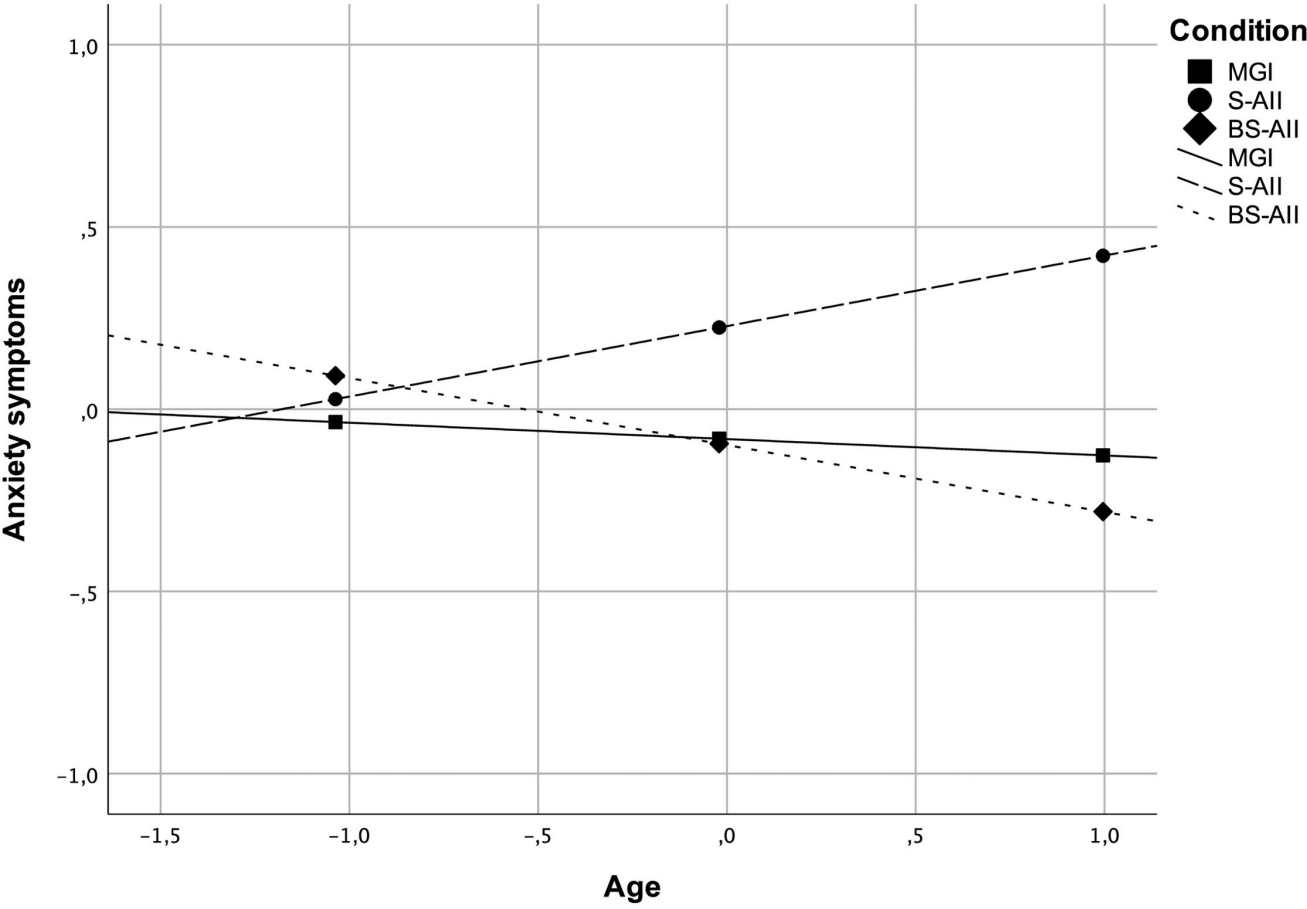
Moderating effects of age between the group assignment and anxiety symptoms at post-intervention (T2). Both the *x*- and *y*-axis represent standardized values.

**Figure 3:**
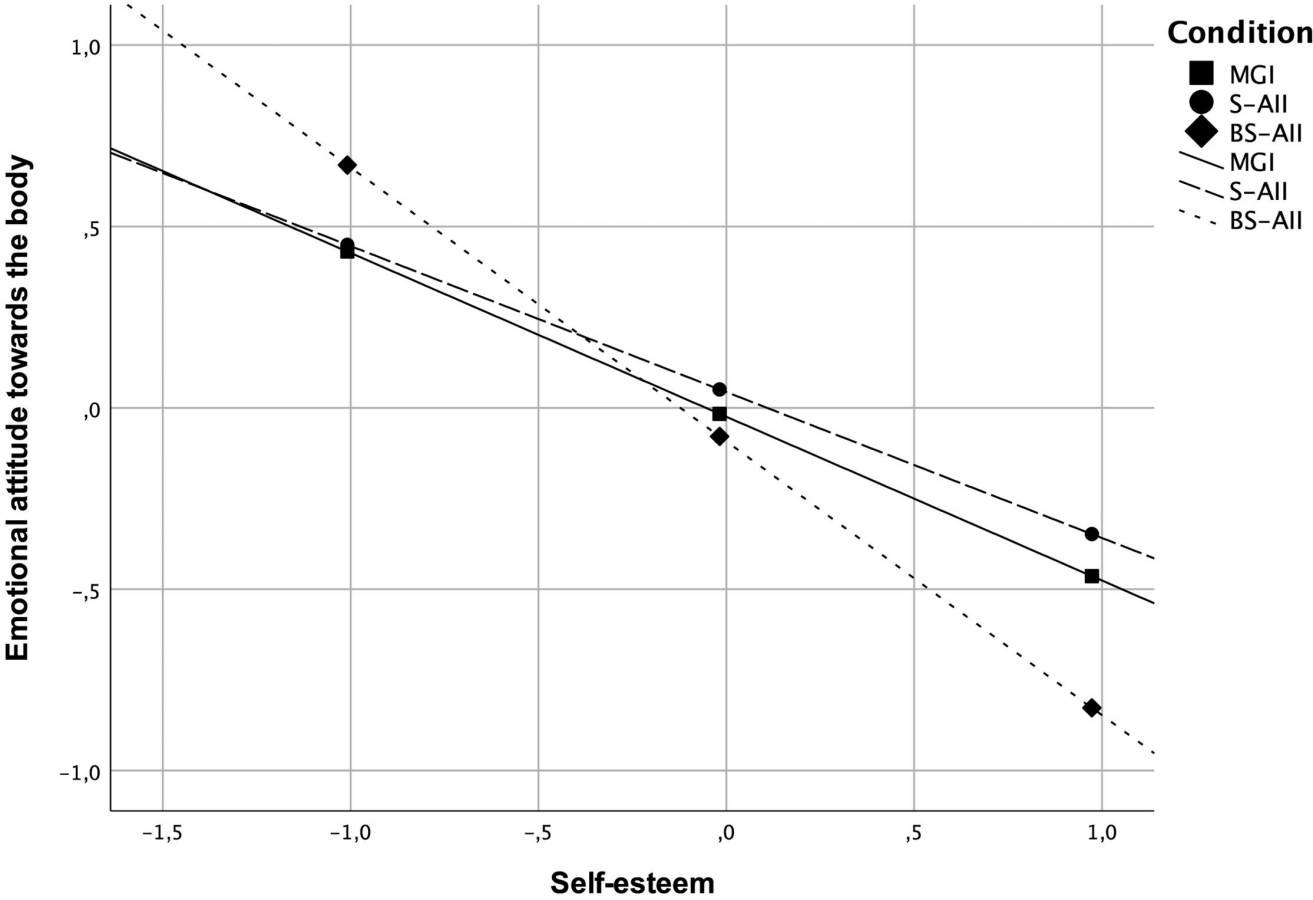
Moderating effects of self-esteem between the group assignment and negative emotional attitude toward the body at post-intervention (T2). Both the *x*- and *y*-axis represent standardized values.

All baseline characteristics were explored to test potential moderators of the intervention effects. Nonetheless, analyses revealed only two significant (*p* < 0.05) moderating effects regarding the outcomes at post-intervention (T2). In the first case, age moderated effects between the group assignment and anxiety symptoms at T2 (see Table 4). It was found that for individuals at an older age, the S-AII intervention compared to the BS-AII resulted in higher levels of anxiety symptoms at post-intervention (*t* = −2.64, *p* = 0.009) (see Figure 2, Table 5). In the second case, self-esteem was found to moderate effects between the group assignment and emotional attitude toward the body at T2 (see Table 4). It was found that for individuals with higher self-esteem, the BS-AII intervention compared to the S-AII resulted in significantly lower levels of negative emotional attitude toward the body at post-intervention (*t* = −2.12, *p* = 0.035) (see Figure 3, Table 5).

**Table 4:** Age and self-esteem as moderators of the intervention effects.

**Moderator**	**Anxiety**				**Emotional attitude toward the body**			
	**Highest order unconditional interaction**	**Coeff. (SE)**	***t***	***p***	**Highest order unconditional interaction**	**Coeff. (SE)**	***t***	***p***
**Age**	*F*_(2, 181)_ = 2.17, *p* = 0.117				*F*_(2, 181)_ = 0.04, *p* = 0.959			
Interaction 1		0.05 (0.15)	0.33	0.741		−0.04 (0.15)	−0.26	0.794
Interaction 2		–0.38 (0.18)	–2.08	0.039		−0.02 (0.19)	−0.10	0.922
**Self-esteem**	*F*_(2, 181)_ = 0.10, *p* = 0.904				*F*_(2, 181)_ = 3.09 *p* = 0.048			
Interaction 1		0.02 (0.15)	0.12	0.903		−0.13 (0.13)	−0.96	0.337
Interaction 2		0.08 (0.18)	0.43	0.668		–0.35 (0.16)	–2.28	0.024

*Interaction 1 – interaction between group (coded as MGI vs. S-AII, BS-AII) and moderator variable; Interaction 2 – interaction between group (coded as S-AII vs. BS-AII) and moderator variable; BS-AII, body-related self-affirming implementation intention; MGI, mere goal intention condition; S-AII, self-affirming implementation intention condition*.

**Table 5:** Conditional effects of the group at values of the tested moderators.

**Anxiety**				**Emotional attitude toward the body**			
**Moderator**	**Coeff. (SE)**	***t***	***p***	**Moderator**	**Coeff. (SE)**	***t***	***p***
**Age**				**Self-esteem**	
*Low*				*Low*	
MGI vs. S-AII, BS-AII	0.10 (0.22)	0.43	0.665	MGI vs. S-AII, BS-AII	0.13 (0.19)	0.68	0.501
S-AII vs. BS-AII	0.06 (0.25)	0.26	0.799	S-AII vs. BS-AII	0.22 (0.21)	1.05	0.295
*Intermediate*				*Intermediate*	
MGI vs. S-AII, BS-AII	0.15 (0.15)	0.96	0.341	MGI vs. S-AII, BS-AII	0.01 (0.13)	0.02	0.985
S-AII vs. BS-AII	−0.32 (0.18)	−1.75	0.083	S-AII vs. BS-AII	−0.13 (0.15)	−0.84	0.402
*High*				*High*	
MGI vs. S-AII, BS-AII	0.20 (0.21)	0.92	0.359	MGI vs. S-AII, BS-AII	−0.13 (0.18)	−0.70	0.490
S-AII vs. BS-AII	–0.70 (0.27)	–2.64	0.009	S-AII vs. BS-AII	–0.48 (0.23)	–2.12	0.035

*S-AII, self-affirming implementation intention condition; BS-AII, body-related self-affirming implementation intention; MGI, mere goal intention condition. Values of the moderator: high (one standard deviation above the mean), intermediate (at the mean), and low (one standard deviation below the mean)*.

## Discussion

Given previous successful attempts of adopting self-affirming implementation intentions that have shown short-term beneficial effects on mental health [e.g., (15–17)] and well-being (9, 11), this study aimed to investigate whether these effects can be further enhanced and could be long-lasting. To accomplish these goals, two specific strategies were adopted and tested within RCT with a fully factorial design. First, modifications of S-AII aiming to strengthen the element of specificity of the intervention (i.e., to body-and skin-related issues) were supplied. It was hypothesized that a critical situation, in the *if*-part of if-then plans with self-affirming cognitions, being specifically defined to body-related issues could further strengthen the effectiveness, enabling both more adjusted self-regulation and more impactful effects of self-affirming thoughts for a community of adults with psoriasis. Second, a booster component as a strategy typically utilized for optimizing beneficial long-term effects of psychological intervention was tested, predicting that the intervention conditions augmented with the booster session would yield much better mental health outcomes assessed at the final study point (i.e., at 1-month follow-up). Contrary to these expectations, there were no significant differences across the groups on mental health, well-being, and secondary outcomes both at post-intervention and at the 1-month follow-up, nor with the control condition forming mere goal intentions as well. Possible explanations for these findings are discussed in the following.

To begin with, some moderation effects were identified through exploratory analyses, pointing to possible boundary conditions of self-affirmation interventions. Two interesting effects emerged. It was found that for individuals at an older age, the S-AII intervention compared to the BS-AII resulted in higher levels of anxiety symptoms at post-intervention. The results suggest that people at an older age benefit much less from being instructed to make general if-then plans with self-affirming cognitions than from those directly pre-defined (i.e., if-then plans that were specifically directed to body-related issues). In the second case, self-esteem was found to moderate the effect between the group assignment and emotional attitude toward the body. It is especially remarkable in light of research on risk factors affecting mental health in psoriasis showing that in patients with psoriasis, basic coping strategies (e.g., emotion-oriented) are secondary to other factors such as low self-esteem [c.f. (53)]. For individuals with higher self-esteem, the BS-AII intervention compared to the standard S-AII resulted in comparably lower levels of negative emotional attitude toward the body at post-intervention, pointing that the element of specificity for those individuals worked better than the standard S-AII. These results also suggest that although self-affirmations benefit certain individuals (i.e., with high self-esteem), they could backfire for the very people who may need them the most. More generally, it seems self-affirming cognitions indeed make additional self-resources more salient, but only for those who have accessibility to them. Self-affirmation as such clearly is not a universal path for improvement in functioning, and as shown not always yields positive effects.

Going back to the theoretical model (3), self-esteem indeed may affect how people respond to self-affirming cognitions because self-affirmation changes the accessibility of alternative, positive identities rather than boosts, inflates, or repairs dispositional self-esteem [c.f. (1, 4, 5)]. Therefore, although tentative, the results suggest that self-affirmations (though positive) could backfire for the very people who as assumed need them the most. For people with low self-esteem, self-affirming cognitions may be highly discrepant from one's initial beliefs and one's self-view, provoking counterarguing and shifting the salience from positive to negative thought domains [c.f. (54–56)]. Of note, these findings stand in with research on positive messages intended to promote positive body image. The results showed their potential to decrease women's body satisfaction priming negative body-related thoughts, particularly for women who believe they are unattractive [c.f. (12)]. Correspondingly, positive self-statements seem to provide a boost only to individuals with relatively high self-esteem/positive self-image, that is, those who ordinarily feel quite good about themselves already. Otherwise, these potentially positive statements, as shown, may stand a risk of undesirable effects. Generating positive self-statements may highlight the discrepancy between people's perceived deficiency and the preferred standard they would like to meet. The discrepancy between their actual and ideal selves as a result becomes more salient, which makes them feel worse [see e.g., (57)]. In line with ‘person-activity fit' concept from the positive-activity model [(58, 59); see also (10)], these findings broadly mirror that certain types of activities could be good for certain types of people, but not for others.

Considering other possible explanations the role of awareness in the process of self-affirmation has to be noted. Researchers have argued that a heightened awareness of an act of self-affirmation in the face of self-evaluative threat could lead people to link the affirmation to the threatened domain rather than broadening their perspective on the threat (4, 60). If people perceive that they are engaged in an emotion regulation task and/or a stress-reduction exercise, they may be more aware of their stressors and negative self-views rather than their self-resources, personal values, or important relationships that should be made salient by the act of self-affirmation. It may be thus the case that with awareness of the impact of self-affirmation, effects are attenuated; and, even subtle issues may be important here (60). For example, it has been shown that when participants were told that the affirmation is expected to benefit them (or they simply were aware of a connection between the task and the outcome measure), its impact was diminished [(61); for a review, see (5, 60)]. Relatedly, it seems that the key to effective affirmation interventions may lie in the subtle manner of their delivery, along with the minimalism of their administration. More transparent (or recursive) interventions may raise awareness, resulting in diminished effectiveness; it could be thus one of the possible determinants of the lack of effectiveness in this study. This insight has therefore important implications for further efforts to apply the theory in intervention settings.

The issue of heightened awareness of self-affirmation processes is also related to the question of types of self-affirmations that can lead to disappointing effects. Research has found that same-domain affirmations exacerbate self-dissonance, whereas alternative domain affirmations reduce it [e.g., (54, 62)]. Same-domain affirmations may reinforce a focus squarely on the threatened domain [c.f. (4)], leading intervention to be ineffective, and even producing negative-enhancing effects. It may be that affirming those identities in self, which are not only different but in particular conceptually unrelated or strictly differentiated from the threatened domain, may yield the most beneficial effects. Future research on S-AII is therefore encouraged to apply this nuanced prediction, which can provide much deeper insight into the nature of self-affirming effects. Additionally, for self-affirmation to yield benefits it may be that the presence of resources to support significant change is needed—some infrastructure or other instrumental content to support sustained positive action (e.g., through behavioral activation) [c.f. (8)]. Because self-affirmation prompts people to reflect on their values, strengths, and/or most important relationships, without real action in some instances it can only make salient the discrepancy between one's currently perceived deficiency and the ideal standard to accomplish. In the absence of opportunities for self-advancement, it may be that self-affirmation could have a limited benefit. It would be worthwhile for future studies to investigate whether providing more resources to support active change could yield consistently larger self-affirmation benefits. It is particularly interesting when the main effect of time is taken into consideration in this study, which indicates that also simply setting a goal intention, as willful direction, initiated positive processes of change. It may be the case that in contrast to S-AII/BS-AII, the formation of goal intentions in terms of behaviors/outcomes/desired future (e.g., “I want to start working out;” “I want to spend more time with my family”) resulted in more focus on action relative to thoughts and could initiate behavioral activation and more instrumental strategies. Consequently, these findings warrant future research.

### Strengths and Limitations

These findings are drawn from the pre-registered RCT with a full factorial design. The strengths of this study also include using a control group matched to the target condition.^4^ Notably, the study has sufficient (assumed) statistical power, which cannot explain the non-significant (main) results,^5^ though it would be the easiest way of explanation. Moreover, the adopted statistical approach (i.e., LMM) enabled the use of all data of each participant in parameter estimation and significance testing, so that the main analyses were performed using the data of all randomized participants, producing more reliable estimates [c.f. (63)]. Moreover, self-affirming implementation intentions—the adopted means of self-affirmation in this study, though differing by design from typical self-affirmation writing exercises [see (20)], was successfully tested in past research [e.g., (11, 16, 17)], which also *per se* cannot constitute an explanation for null results in this study.^6^ With a broader perspective, as provided in the discussion above, these null results estimated based on the confirmatory study with sufficient power for the experimental factorial design offer an important contribution to knowledge building and further research.

There are, however, several limitations in this study that should be thoroughly considered. Participants in this study represented only a segment of the community population that was interested and engaged to actively respond to the study. Moreover, the sample was not gender-balanced, and women were in majority. Additionally, more strict inclusion criteria to be included in the trial of having at least moderate levels of anxiety and depressive symptoms were not adopted, so conclusions are limited to a sub-clinical sample of participants being at risk/with elevated symptoms of depression and anxiety. On the other hand, exclusionary practices (as insufficient or too severe symptoms) would eliminate a large proportion of a representative cohort of individuals from trial participation, limiting the generalizability of the findings, reducing the confidence that findings can be translated into real-world settings and, crucially, resulting in reported overly larger (overestimated) effects. The final limitation to note concerns using solely self-reported measures. Although the scales used are well-validated and research has demonstrated their sensitivity to change, future investigations on S-AII are encouraged to apply an ecological momentary assessment approach for outcome measures to better evaluate intervention effectiveness. This approach offers more sensitivity in detecting changes; moreover, its higher precision in measuring intervention effectiveness allows determining whether intervention effects are robust or varying over time. Notably, it can help set the time at which the intervention effects have leveled off or diminished and booster sessions would be needed to strengthen and/or expand intervention effects [c.f. (64, 65)].

## Conclusion

Self-affirmation interventions should be further investigated and optimized before they can be broadly implemented in real-life contexts, especially to prevent backfiring and negative-enhancing effects. This suggestion is clearly mirrored in the realm of the current findings. As shown, self-affirmation might produce null effects or even backfire—a topic of considerable current interest [see (5, 8, 66)]. Although self-affirmation interventions have shown positive effects across the literature, the presence of null and negative findings, reported also in previous studies [e.g., (12, 13)], suggests potential moderators and substantial boundary conditions that need to be addressed in a deeper and more systematic manner.

## Data Availability Statement

The raw data supporting the conclusions of this article will be made available by the author, without undue reservation.

## Ethics Statement

The study was performed with ethical approval granted by the University's Institutional Review Board and in accordance with the ethical standards as laid down in the 1964 Declaration of Helsinki and its later amendments or comparable ethical standards. Informed consent was obtained from all individual participants included in the study.

## Author Contributions

The author confirms being the sole contributor of this work and has approved it for publication.

## Funding

Research reported in this publication was supported by the National Science Center in Poland under research grant 2017/25/N/HS6/01319. The funding body had no role in the design and conduct of the study; collection, management, analysis, and interpretation of the data; preparation, review, or approval of the manuscript; and decision to submit the manuscript for publication.

## Conflict of Interest

The author declares that the research was conducted in the absence of any commercial or financial relationships that could be construed as a potential conflict of interest.

## Publisher's Note

All claims expressed in this article are solely those of the authors and do not necessarily represent those of their affiliated organizations, or those of the publisher, the editors and the reviewers. Any product that may be evaluated in this article, or claim that may be made by its manufacturer, is not guaranteed or endorsed by the publisher.

## Footnotes

^1^Łakuta P. Turning to better mental health through self-affirming implementation intentions: mediators and moderators of intervention effects of a randomized controlled trial [Manuscript submitted for publication]. (under review).

^2^The full study protocol can be accessed at https://osf.io/nd9ce.

^3^All the results were very similar for the LMM models on outcomes at post-intervention (T2) only, testing effects of time, condition, and their interactions.

^4^The control group was matched to allow the effect of the self-affirmation interventions to be examined above and beyond the effect of simply setting goal intention, so any conclusions should be seen relative to the efficacy of that control condition (rather than relative to no treatment).

^5^However, it should be kept in mind that to estimate smaller differences in the intervention and for moderation effects especially, larger sample size would be advisable (c.f. a priori power analysis).

^6^Lack of the manipulation check, however, still raises the possibility that the observed null results may be a failure to induce self-affirmation, rather than a failure of self-affirmation *per se* to affect mental health. On the other hand, such manipulation checks come with major problems (they may amplify or undo the effects of a manipulation). Evaluating means of successfully inducing self-affirmation (and the issue of proper assessment methods for manipulation checks) decisively needs further research.
