# Supplementary material for: A Factorial Randomized Controlled Trial of Implementation-Intention-Based Self-Affirmation Interventions: Findings on Depression, Anxiety, and Well-being in Adults With Psoriasis
Source: Front Psychiatry. 2022 Mar 18;13:795055. doi: 10.3389/fpsyt.2022.795055 (PMC8971365; doi:10.3389/fpsyt.2022.795055)
Supplement: Supplementary file 1 [file Data_Sheet_1.docx]

Supplementary Material

A factorial randomized controlled trial of implementation-intention-based self-affirmation interventions: Findings on depression, anxiety, and well-being in adults with psoriasis

**Patryk Łakuta***

*** Corresponding Author: plakuta@swps.edu.pl**

|  |  |  | **Depression** | | | **Anxiety** | | | **Well-being** | | |
| --- | --- | --- | --- | --- | --- | --- | --- | --- | --- | --- | --- |
| Booster | Group | Time | *M* | *SE* | 95% CI | *M* | *SE* | 95% CI | *M* | *SE* | 95% CI |
| yes | MGI | 1 | 10.11 | 1.07 | 8.00;  12.22 | 10.83 | 1.00 | 8.87;  12.80 | 29.6 | 2.90 | 23.9;  35.3 |
| no | MGI | 1 | 9.85 | 0.95 | 7.99;  11.71 | 10.84 | 0.88 | 9.10;  12.57 | 32.4 | 2.56 | 27.3;  37.4 |
| yes | S-AII | 1 | 10.08 | 1.06 | 8.00;  12.16 | 10.54 | 0.99 | 8.61;  12.48 | 29.4 | 2.86 | 23.8;  35.0 |
| no | S-AII | 1 | 10.16 | 0.95 | 8.30;  12.03 | 10.94 | 0.88 | 9.20;  12.67 | 28.3 | 2.56 | 23.2;  33.3 |
| yes | BS-AII | 1 | 8.48 | 1.12 | 6.28;  10.67 | 8.98 | 1.04 | 6.93;  11.03 | 31.1 | 3.02 | 25.1;  37.0 |
| no | BS-AII | 1 | 9.13 | 0.92 | 7.31;  10.94 | 10.10 | 0.86 | 8.41;  11.79 | 30.2 | 2.50 | 25.3;  35.1 |
| yes | MGI | 2 | 8.33 | 1.07 | 6.22;  10.44 | 8.27 | 1.00 | 6.30;  10.23 | 32.0 | 2.90 | 26.3;  37.7 |
| no | MGI | 2 | 8.61 | 1.00 | 6.65;  10.58 | 7.91 | 0.94 | 6.06;  9.76 | 32.9 | 2.66 | 27.7;  38.1 |
| yes | S-AII | 2 | 9.44 | 1.06 | 7.37;  11.52 | 10.12 | 0.99 | 8.18;  12.06 | 31.1 | 2.86 | 25.4;  36.7 |
| no | S-AII | 2 | 8.10 | 1.03 | 6.08;  10.11 | 9.13 | 0.97 | 7.22;  11.04 | 32.0 | 2.71 | 26.6;  37.3 |
| yes | BS-AII | 2 | 7.48 | 1.12 | 5.28;  9.67 | 8.08 | 1.04 | 6.03;  10.13 | 31.3 | 3.02 | 25.3;  37.2 |
| no | BS-AII | 2 | 7.73 | 1.05 | 5.66;  9.80 | 7.95 | 1.01 | 5.97;  9.92 | 34.4 | 2.76 | 28.9;  39.8 |
| yes | MGI | 3 | 8.14 | 1.07 | 6.03;  10.25 | 7.36 | 1.00 | 5.40;  9.33 | 35.5 | 2.90 | 29.7;  41.2 |
| no | MGI | 3 | 8.76 | 1.01 | 6.78;  10.75 | 7.76 | 0.95 | 5.89;  9.63 | 33.1 | 2.68 | 27.8;  38.3 |
| yes | S-AII | 3 | 9.46 | 1.07 | 7.36;  11.57 | 7.79 | 1.00 | 5.82;  9.76 | 31.9 | 2.88 | 26.2;  37.5 |
| no | S-AII | 3 | 7.97 | 1.04 | 5.92;  10.01 | 7.47 | 0.99 | 5.52;  9.41 | 33.3 | 2.74 | 27.9;  38.7 |
| yes | BS-AII | 3 | 6.44 | 1.16 | 4.17;  8.72 | 6.85 | 1.09 | 4.72;  8.99 | 36.2 | 3.09 | 30.2;  42.3 |
| no | BS-AII | 3 | 7.69 | 1.05 | 5.62;  9.76 | 7.84 | 1.01 | 5.87;  9.82 | 33.9 | 2.76 | 28.4;  39.3 |

**Table A.** Estimated marginal means with 95% confidence intervals on primary outcomes across all study groups based on LMMs.

*Note*. S-AII = self-affirming implementation intention condition; BS-AII = body-related self-affirming implementation intention;
MGI = mere goal intention condition.

**Table B.** Estimated marginal means with 95% confidence intervals on secondary outcomes across all study groups based on LMMs.

|  |  |  | **Emotional attitude towards the body** | | | **Positive self-directed feelings** | | | **Positive other-directed feelings** | | |
| --- | --- | --- | --- | --- | --- | --- | --- | --- | --- | --- | --- |
| Booster | Group | Time | *M* | *SE* | 95% CI | *M* | *SE* | 95% CI | *M* | *SE* | 95% CI |
| yes | MGI | 1 | 46.0 | 2.27 | 41.6;  50.5 | 9.64 | 0.76 | 8.14;  11.14 | 12.1 | 0.68 | 10.7;  13.4 |
| no | MGI | 1 | 41.6 | 2.00 | 37.6;  45.5 | 9.40 | 0.67 | 8.07;  10.72 | 12.9 | 0.60 | 11.7;  14.1 |
| yes | S-AII | 1 | 43.5 | 2.23 | 39.1;  47.9 | 9.45 | 0.75 | 7.97;  10.93 | 12.6 | 0.67 | 11.3;  13.9 |
| no | S-AII | 1 | 45.1 | 2.00 | 41.1;  49.0 | 8.03 | 0.67 | 6.70;  9.35 | 13.2 | 0.60 | 12.0;  14.4 |
| yes | BS-AII | 1 | 45.7 | 2.36 | 41.1;  50.4 | 9.20 | 0.80 | 7.63  10.76 | 13.5 | 0.71 | 12.1;  14.9 |
| no | BS-AII | 1 | 42.5 | 1.95 | 38.7;  46.3 | 8.88 | 0.66 | 7.58;  10.17 | 11.7 | 0.59 | 10.6;  12.9 |
| yes | MGI | 2 | 42.9 | 2.27 | 38.5;  47.4 | 10.23 | 0.76 | 8.73;  11.73 | 12.4 | 0.68 | 11.0;  13.7 |
| no | MGI | 2 | 38.9 | 2.09 | 34.8;  43.0 | 9.65 | 0.71 | 8.25;  11.04 | 12.6 | 0.64 | 11.3;  13.8 |
| yes | S-AII | 2 | 42.2 | 2.23 | 37.8;  46.6 | 10.00 | 0.75 | 8.52;  11.48 | 13.0 | 0.67 | 11.7;  14.4 |
| no | S-AII | 2 | 42.2 | 2.13 | 38.1;  46.4 | 9.28 | 0.73 | 7.85;  10.71 | 13.6 | 0.66 | 12.3;  14.9 |
| yes | BS-AII | 2 | 42.5 | 2.36 | 37.9;  47.2 | 9.20 | 0.80 | 7.63;  10.76 | 13.3 | 0.71 | 11.9;  14.7 |
| no | BS-AII | 2 | 41.0 | 2.16 | 36.7;  45.2 | 10.54 | 0.74 | 9.08;  12.00 | 12.2 | 0.67 | 10.9;  13.6 |
| yes | MGI | 3 | 37.7 | 2.27 | 33.3;  42.2 | 10.73 | 0.76 | 9.23;  12.23 | 12.2 | 0.68 | 10.9;  13.6 |
| no | MGI | 3 | 37.0 | 2.10 | 32.8;  41.1 | 9.96 | 0.72 | 8.56;  11.37 | 12.4 | 0.64 | 11.1;  13.7 |
| yes | S-AII | 3 | 41.4 | 2.25 | 36.9;  45.8 | 10.18 | 0.76 | 8.68;  11.68 | 12.4 | 0.68 | 11.1;  13.8 |
| no | S-AII | 3 | 39.8 | 2.15 | 35.5;  44.0 | 10.93 | 0.74 | 9.48;  12.38 | 13.3 | 0.67 | 12.0;  14.7 |
| yes | BS-AII | 3 | 40.6 | 2.42 | 35.9;  45.4 | 10.95 | 0.82 | 9.34;  12.56 | 14.0 | 0.74 | 12.5;  15.4 |
| no | BS-AII | 3 | 41.8 | 2.16 | 37.6;  46.1 | 11.11 | 0.74 | 9.65;  12.57 | 12.3 | 0.67 | 10.9;  13.6 |

*Note*. S-AII = self-affirming implementation intention condition; BS-AII = body-related self-affirming implementation intention;
MGI = mere goal intention condition.
